# The *TCF7L2 *locus and type 1 diabetes

**DOI:** 10.1186/1471-2350-8-51

**Published:** 2007-08-03

**Authors:** Hui-Qi Qu, Constantin Polychronakos

**Affiliations:** 1 Endocrine Genetics Lab, The McGill University Health Center (Montreal Children's Hospital), Montréal, Québec, Canada; 2 The McGill University Health Center (Montreal Children's Hospital), 2300 Tupper, Montréal, Québec, H3H 1P3, Canada

## Abstract

**Background:**

*TCF7L2 *belongs to a subfamily of TCF7-like HMG box-containing transcription factors, and maps to human chromosome 10q25.3. A recent study identified genetic association of type 2 diabetes (T2D) with this gene, correlated with diminished insulin secretion. This study aimed to investigate the possibility of genetic association between *TCF7L2 *and type 1 diabetes (T1D).

**Methods:**

The SNP most significantly associated with T2D, rs7903146, was genotyped in 886 T1D nuclear family trios with ethnic backgrounds of mixed European descent.

**Results:**

This study found no T1D association with, and no age-of-onset effect from rs7903146.

**Conclusion:**

This study suggests that a T2D mechanism mediated by *TCF7L2 *does not participate in the etiology of T1D.

## Background

Recently, a new type 2 diabetes (T2D) susceptibility gene, transcription factor 7-like 2 (*TCF7L2*), was identified[1]. Subsequently, many studies confirmed this novel association[2-12], which accounts for the highest T2D risk confirmed to date. *TCF7L2 *belongs to a subfamily of TCF7-like HMG box-containing transcription factors, and maps to human chromosome 10q25.3[13]. It is a component of the Wnt signaling pathway, and participates in the tissue-specific regulation of expression of proglucagon gene, which has been shown critical in blood glucose homeostasis[14]. Because of the effects on blood glucose homeostasis, the *TCF7L2 *gene variation may also be important in type 1 diabetes (T1D). To investigate the possibility of this association, we genotyped the marker most significantly associated with T2D, the intronic SNP rs7903146, in our T1D family collection.

## Methods

### Subjects

Genomic DNA (gDNA) was obtained after informed consent from T1D-affected subjects and their two parents (886 trios or 2,658 individuals after removing families with Mendelian discrepancies at multiple independent SNPs). The Research Ethics Board of the Montreal Children's Hospital and other participating centers approved the study. Ethnic backgrounds were of mixed European descent, with the largest single group being of Quebec French-Canadian origin (40% of the total collection).

### SNP Genotyping

The SNP rs7903146 was genotyped by the Sequenom MassARRAY system (Sequenom, San Diego CA, USA). Primers for PCR and MassEXTEND reaction to detect sequence differences at the single nucleotide level were designed using Assay Design software. PCR primers: forward, 5'-ACGTTGGATGGGTGCCTCATACGGCAATTA-3'; reverse, 5'-ACGTTGGATGTCTCTGCCTCAAAACCTAGC-3'. The extension primer, 5'-AGAGCTAAGCACTTTTTAGATA-3'. We amplified 2.5 ng of gDNA in a 5 μl volume. After dephosphorylation of unincorporated dNTPs, allele-specific primer extension reaction was performed to generate different sizes of extension products corresponding to each allele by the Sequenom iPLEX™ technology. Samples were conditioned to remove extraneous salts and genotypes were called based on the presence of different sizes of extension products by the Matrix Assisted Laser Desorption Ionization Time Of Flight (MALDI-TOF) mass spectrometry technology[15]. The call rate of this SNP is 99.6% and the Mendelian error rate is 0.2%.

### Statistics

Hardy-Weinberg equilibrium (HWE) of the genotype distribution in parents of the T1D families was tested by the following equation.

χ2=(a+b+c)(b2−4ac)2(2a+b)2(b+2c)2,
 MathType@MTEF@5@5@+=feaafiart1ev1aaatCvAUfKttLearuWrP9MDH5MBPbIqV92AaeXatLxBI9gBaebbnrfifHhDYfgasaacH8akY=wiFfYdH8Gipec8Eeeu0xXdbba9frFj0=OqFfea0dXdd9vqai=hGuQ8kuc9pgc9s8qqaq=dirpe0xb9q8qiLsFr0=vr0=vr0dc8meaabaqaciaacaGaaeqabaqabeGadaaakeaacqaHhpWydaahaaWcbeqaaiabikdaYaaakiabg2da9maalaaabaGaeiikaGIaeeyyaeMaey4kaSIaeeOyaiMaey4kaSIaee4yamMaeiykaKIaeiikaGIaeeOyai2aaWbaaSqabeaacqaIYaGmaaGccqGHsislcqaI0aancqqGHbqycqqGJbWycqGGPaqkdaahaaWcbeqaaiabikdaYaaaaOqaaiabcIcaOiabikdaYiabbggaHjabgUcaRiabbkgaIjabcMcaPmaaCaaaleqabaGaeGOmaidaaOGaeiikaGIaeeOyaiMaey4kaSIaeGOmaiJaee4yamMaeiykaKYaaWbaaSqabeaacqaIYaGmaaaaaOGaeiilaWcaaa@512B@

ν = 1; a, b and c are the frequencies of, respectively, the C/C, C/T or T/T genotypes.

Transmission disequilibrium test (TDT) was performed by the Haploview software[16]. The age effect among different genotypes was tested by both ANOVA and the nonparametric Kruskal-Wallis Test.

## Results and discussion

The minor allele T of rs7903146 has a frequency of 0.303 in the parents of the T1D families, which is similar to the frequencies of control groups in the T2D studies. The genotype distribution in the parents was in HWE (χ^2 ^= 0.0, ν = 1, p = 0.972), compatible with the absence of stratification, admixture or technical artifacts. Our study found no T1D association from rs7903146. The transmission ratio T/C = 369/342 (**χ^2 ^**= 1.0, p = 0.311).

**Figure 1:**
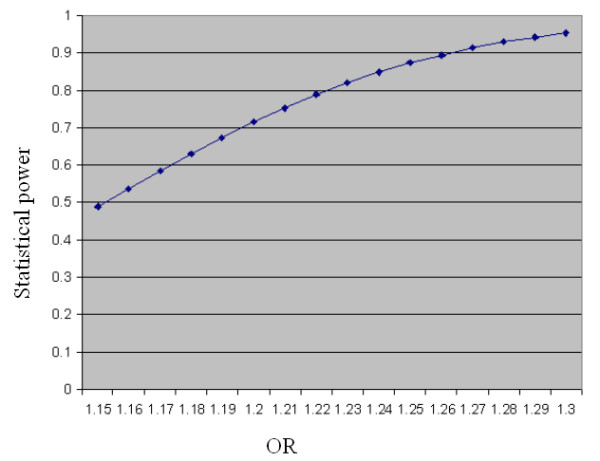
The statistical power of this study to detect a genetic association with the minor allele frequency of 0.303 at α = 0.05 level. X-axis: the OR value; Y-axis: the statistical power.

For a SNP with the minor allele frequency of 0.303, the statistical power at α = 0.05 level of our family-based association study on 886 affected nuclear family trios is shown in Figure 1. Our study has a statistic power of > 80% at α = 0.05 level to detect a genetic association with an effect size as low as OR = 1.23. Besides the *HLA *region[17], previously confirmed T1D associations have been replicable in our dataset, including the weak effect from the SNP rs231775 on the *CTLA4 *gene(Table 1). Considering the important role of *TCF7L2 *in glucose homeostasis, one possibility that needs to be excluded is that the *TCF7L2 *gene variation might accelerate the onset of clinical symptoms of T1D. We compared the age-of-onset of different genotypes of the *TCF7L2 *rs7903146 (Table 2). We found no age-of-onset effect from the *TCF7L2 *variation. Therefore, our result suggests that the genetic variation of *TCF7L2 *has no obvious effect on T1D.

**Table 1:** Transmission disequilibrium tests of the SNPs in the *INS*, *PTPN22*, and *CTLA4 *gene in the T1D trio set used in this study

**gene**	**dbSNP ID**	**minor allele (frequency)**	Transmission ratio*	**χ**^2 ^(p value)	OR (95% CI)
***INS***	rs689	T (0.207)	186:387	70.5 (4.58 × 10^-17^)	0.48 (0.40, 0.57)
***PTPN22***	rs2476601	A (0.126)	251:123	43.8 (3.62 × 10^-11^)	2.04 (1.64, 2.53)
***CTLA4***	rs231775	G (0.387)	443:382	4.5 (0.034)	1.16 (1.01, 1.33)
***CTLA4***	rs5742909	T (0.090)	137:178	5.3 (0.021)	0.77 (0.62, 0.96)

* Minor allele transmitted : untransmitted.

**Table 2:** The age-of-onset of T1D of three rs7903146 genotypes

genotype	number	average age-of-onset (x¯ MathType@MTEF@5@5@+=feaafiart1ev1aaatCvAUfKttLearuWrP9MDH5MBPbIqV92AaeXatLxBI9gBaebbnrfifHhDYfgasaacH8akY=wiFfYdH8Gipec8Eeeu0xXdbba9frFj0=OqFfea0dXdd9vqai=hGuQ8kuc9pgc9s8qqaq=dirpe0xb9q8qiLsFr0=vr0=vr0dc8meaabaqaciaacaGaaeqabaqabeGadaaakeaacuqG4baEgaqeaaaa@2E3B@ ± *s*)	Median
C/C	355	8.1 ± 4.3	8.3
C/T	323	8.8 ± 5.6	8.1
T/T	74	8.3 ± 5.6	7.6
F value (p)		F = 1.540 (p = 0.215)	
Kruskal Wallis test		χ^2 ^= 1.427 (p = 0.490)	

*TCF7L2 *encodes a transcription factor, also known as T-cell transcription factor 4 (TCF4). TCF4 acts through binding with β-catenin as a complex, and consequently activates the expression of TCF4-regulated genes[18,19]. How the *TCF7L2 *variation changes the gene function is unclear yet. TCF4 is a 596aa peptide (NP_110383.1), which contains two conserved domains, the CTNNB1_binding domain and the SOX-TCF_HMG-box domain. The CTNNB1_binding domain locates from the 1^st ^to the 236^th ^amino acid, which binds to β-catenin and forms the active complex. The SOX-TCF_HMG-box domain locates from the 330^th ^to the 397^th ^amino acid, which binds to specific DNA motif and regulates gene transcription. Sequencing of all the exons of *TCF7L2 *in 184 individuals (93 T2D cases and 91 controls) has found no nonsynonymous SNP (nsSNP)[1]. The possibility of protein-sequence polymorphism can be excluded as the basis for the T2D susceptibility. Therefore, the T2D susceptibility should be from the change of gene expression level resulting from regulatory genetic variation. The *TCF7L2 *gene spans ~216 kb and ~750 SNPs have been identified in this region. As shown by the international HapMap project data, *TCF7L2 *spans several linkage disequilibrium (LD) blocks in Europeans[20]. The T2D-associated SNPs are from the largest LD block spanning ~65 kb, which starts from the middle of intron 3, and ends at the first seventh of intron 4[1,21]. The genetic regulation on *TCF7L2 *expression can be from the change of mRNA processing or alternative splicing from a variation in the gene intronic region. There is no published information on any such effects; unpublished data from our lab (Marchand et al., work in progress) has failed to find allelic effects on mRNA levels or relative abundance of splicing isoforms in the few tissues examined to date.

T2D results from a progressive insulin secretory defect on a background of insulin resistance[22]. However, the role of *TCF7L2 *in the T2D mechanism is unclear. Present studies show contradictory results on the effect of the *TCF7L2 *gene variation on insulin secretion and insulin sensitivity. Florez JC, et al, showed that the T2D-predisposing haplotype correlates with diminished insulin secretion but has no effect on insulin response[3]. On the other hand, Elbein SC, et al, showed that the *TCF7L2 *gene variation is correlated with reduced glucose tolerance and insulin sensitivity, but not insulin secretion[23]. In either case, it would have been reasonable to hypothesize that the T2D-risk genotypes at *TCF7L2 *may accelerate the clinical onset of T1D symptoms; none was seen in our study.

## Conclusion

Our study suggests the *TCF7L2 *gene variation does not participate in the etiology of T1D. During the manuscript preparation of this study, another report of no association between T1D and *TCF7L2 *was published, which is concordant with our conclusion[24]. The combined results of both studies clearly show that the genetic susceptibility from the *TCF7L2 *gene variation is a unique mechanism of T2D, and is not shared by T1D.

## Abbreviations

T1D, type 1 diabetes; T2D, type 2 diabetes; *TCF7L2*, transcription factor 7-like 2; *INS*, insulin; SNP, single-nucleotide polymorphism; TDT, Transmission disequilibrium test

## Competing interests

The author(s) declare that they have no competing interests.

## Authors' contributions

HQQ carried out the implementation and analysis, and drafted the manuscript. CP conceived of the study, participated in its design and coordination, and participated in preparation of the manuscript. Both authors approved the final manuscript.

## Pre-publication history

The pre-publication history for this paper can be accessed here:


